# Supplementary material for: Validation of the questionnaire “Pregnancy Vaccine Hesitancy Scale (pVHS)” toward COVID-19 vaccine for Malaysian pregnant women
Source: PeerJ. 2024 Mar 25;12:e17134. doi: 10.7717/peerj.17134 (PMC10977085; doi:10.7717/peerj.17134)
Supplement: Supplemental Information 2 [file peerj-12-17134-s002.docx]

**Skala Keraguan Vaksin COVID-19 dalam kalangan wanita hamil**

| **Skala** | **Sangat tidak setuju** | **Tidak setuju** | **Tidak pasti** | **Setuju** | **Sangat setuju** |
| --- | --- | --- | --- | --- | --- |
|  | 1 | 2 | 3 | 4 | 5 |

Arahan : Tandakan **(√)** pada **SATU** jawapan di dalam kotak skala yang disediakan.

| **Bil** | **Perkara** | **1** | **2** | **3** | **4** | **5** |
| --- | --- | --- | --- | --- | --- | --- |
|  | Vaksin COVID-19 adalah penting untuk kesihatan saya. |  |  |  |  |  |
|  | Vaksin COVID-19 adalah berkesan untuk mengurangkan  keterukan jangkitan. |  |  |  |  |  |
|  | Vaksin COVID-19 adalah penting untuk kesihatan  masyarakat di komuniti saya. |  |  |  |  |  |
|  | Vaksin COVID-19 yang disarankan oleh kerajaan  Malaysia adalah bermanfaat. |  |  |  |  |  |
|  | Maklumat yang saya terima mengenai vaksin COVID-19 dari Kementerian Kesihatan Malaysia (KKM) boleh dipercayai. |  |  |  |  |  |
|  | Menerima suntikan vaksin COVID-19 adalah kaedah yang bagus untuk melindungi saya daripada jangkitan penyakit yang teruk. |  |  |  |  |  |
|  | Secara amnya, saya bersetuju dengan semua saranan Kementerian Kesihatan Malaysia berkaitan vaksin COVID-19. |  |  |  |  |  |
|  | Saya bimbang akan kesan sampingan vaksin yang serius. |  |  |  |  |  |
