# Supplementary material for: Validation of the questionnaire “Pregnancy Vaccine Hesitancy Scale (pVHS)” toward COVID-19 vaccine for Malaysian pregnant women
Source: PeerJ. 2024 Mar 25;12:e17134. doi: 10.7717/peerj.17134 (PMC10977085; doi:10.7717/peerj.17134)
Supplement: Supplemental Information 3 [file peerj-12-17134-s003.docx]

**Permarkahan Skala Keraguan Vaksin COVID-19 dalam kalangan wanita hamil**

| **Skala** | **Sangat tidak setuju** | **Tidak setuju** | **Tidak pasti** | **Setuju** | **Sangat setuju** |
| --- | --- | --- | --- | --- | --- |
|  | 1 | 2 | 3 | 4 | 5 |
| **Markah** | 1 | 2 | 3 | 4 | 5 |

**Markah minima = 8**

**Markah maksima = 40**

**Interpretasi tahap keraguan vaksin: Semakin rendah markah keseluruhan skala adalah semakin tinggi tahap keraguan vaksin.**
